# Complete Genome Sequence of Cotton Leafroll Dwarf Virus Infecting Cotton in Oklahoma, USA

**DOI:** 10.1128/mra.00147-22

**Published:** 2022-06-06

**Authors:** Connor Ferguson, Akhtar Ali

**Affiliations:** a Department of Biological Science, The University of Tulsa, Tulsa, Oklahoma, USA; KU Leuven

## Abstract

Cotton leafroll dwarf virus (CLRDV) (genus *Polerovirus*, family *Luteoviridae*) was first reported in the United States in 2017. In this study, we present a complete genome sequence (5,866 nucleotides) of a CLRDV isolate, EC4, that had been collected during the 2021 cotton growing season in Oklahoma.

## ANNOUNCEMENT

Cotton (Gossypium hirsutism L.) represents a major cash crop in the United States and globally. Cotton leafroll dwarf virus (CLRDV) was first reported in the United States in Alabama during the 2017 growing season (1). Thereafter, it was reported in cotton fields in almost a dozen states (2–12). CLRDV has a positive single-stranded RNA genome of approximately 5,865 nucleotides (13).

CLRDV was first reported in Oklahoma during the 2020 growing season (9) but, to our knowledge, no complete genome of CLRDV has been reported. Therefore, in this work, we sequenced a complete genome sequence of a CLRDV isolate from cotton in Oklahoma.

During the 2021 growing season, 13 cotton leaf samples showing signs of virus infection, including mosaic discoloration and leaf curling, were collected from commercial cotton fields in Beckham County, Oklahoma, and brought to the University of Tulsa. Total RNA was extracted and subjected to reverse transcription (RT)-PCR using CLRDV-specific primers AL674F and AL1407R, as described previously (1). Ten of the 13 total samples were positive, and one positive sample (EC4) was randomly selected for high-throughput sequencing at the Oklahoma State University (OSU) genomics facility using 10 μL total RNA. A plant RNA library kit with Ribo-Zero (Illumina, USA) was used to obtain an indexed plant ribosome-negative sequencing library according to the manufacturer’s instructions. This was followed by shotgun sequencing using the NextSeq 500/550 high-output kit v2.5 (Illumina, USA). The paired-end reads were cleaned of low-quality or adapter sequences using Illumina bcl2fastq2 software v2.2 with default parameters. A total of 18,914,064 trimmed paired-end reads, with an average sequence length of 73.9 bp, were assembled using CLC Genomics Workbench v12.0.3 (Qiagen), resulting in 378,584 contigs. All software parameters were the default settings except that the cutoff limit for nonsignificant sequences was 60 bp, rather than the default 200 bp. The contigs were compared using a BLASTn (NCBI) search against the GenBank nonredundant database.

**FIG 1:**
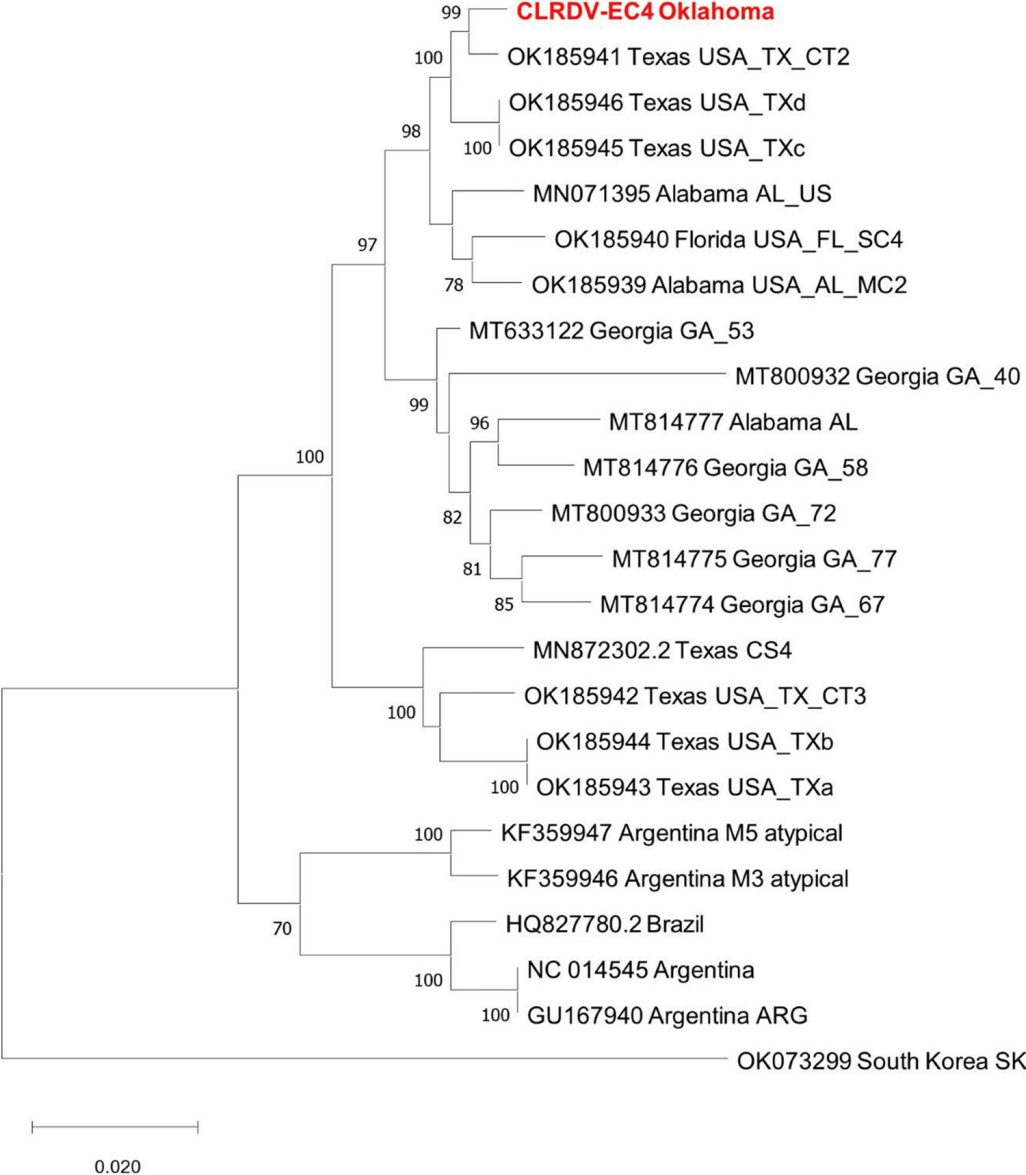
Maximum likelihood phylogenetic tree based on the complete genome nucleotide sequences of CLRDV, obtained using MEGA X with 1,000 bootstraps. Complete genome sequences of all other CLRDV isolates were downloaded from the GenBank nonredundant database. Bootstrap values above 70 are shown on the nodes of the branches of the tree.

A total of 17 contigs (average coverage, 48×) matching CLRDV were obtained; they ranged in length from 78 to 869 bp. A BLASTn (NCBI) search showed that these contigs were not matching a single CLRDV isolate but instead were matching different CLDRV isolates due to their various lengths and genomic locations. The final length of the Illumina extracted partial contig was 5,633 nucleotides. There were missing regions throughout the sequence. The missing regions were obtained by using primers designed in our laboratory, such as the primer targeting the 5′ and 3′ ends of the untranslated region (UTR) (Table 1), or previously reported primers (11, 12) to convert the RNA into cDNA (9). The amplified DNA was either cloned into a plasmid and sequenced at least three times or directly sequenced with ExoSAP-IT reagent (Affymatrix) using Sanger sequencing. Using areas of overlap between the sequences, the Illumina and Sanger sequencing results were combined, resulting in a complete genome of CLRDV isolate EC4, which was 5,866 nucleotides in length. BLASTn (NCBI) searches of the GenBank nonredundant database showed that CLRDV isolate EC4 shared 99.22% nucleotide identity and 98.03% amino acid identity with CLRDV isolate CT2 (GenBank accession number OK185941.1) from Texas (13), which is supported by the MEGA X phylogenetic analysis (Fig. 1).

**TABLE 1:** Primers designed and used in RT-PCR assays to obtain the complete genome of CLRDV isolate EC4

Primer name	Primer sequence (5′ to 3′)	Primer positions (EC4)	Amplification size (bp)	*T_m_* (°C)
CLRDV1F	CGAGCACAACCCATTCCAAA	192–212	623	51
CLRDV1R	CTGCTGGATTTGGCTTTGGT	794–814		
CLRDV2F	CTACGCTTTTGAGACGACCG	1402–1422	1,049	51
CLRDV2R	AAAGACCAGCCGAGAAAGGA	2432–2452		
CLRDV3F	CGTGATCCCAGCTTCCAAAG	4474–4494	1,005	51
CLRDV3R	TTGTCTAGCATCGACCTCCC	5480–5500		
CLRDV5′F	ACAAAAGAACGATAGAGGG	1–19	365	48
CLRDV5′R	CTTGGTGGCTCTTGAAAG	348–365		
CLRDV3′F	TGCTAGACAAGCGTGAT	5507–5523	360	47
CLRDV3′R	ACACCAGAACCCCAGG	5852–5867		

### Data availability.

The complete genome sequence of CLRDV isolate EC4 was deposited in GenBank under accession number OM687235. The raw data have been deposited in the Sequence Read Archive (SRA) under BioProject accession number PRJNA597539 (BioSample accession number SAMN27675195).
